# Preparation and characterization of immobilized mannanase on polyhydroxyalkanoate (PHA)

**DOI:** 10.1186/s40643-025-00886-5

**Published:** 2025-06-25

**Authors:** Zhiyue Men, Yafei Zhang, Zhao Pang, Tao Li, Hening Zhang, Yue Zhou, Ning Hao, Yajian Song, Yu Bai

**Affiliations:** 1 Tianjin Institute of Industrial Biotechnology, Chinese Academy of Sciences, Tianjin, 300308 China; 2 College of Biological Engineering, Tianjin University of Science & Technology, Tianjin, 300457 China; 3 Haihe Laboratory of Synthetic Biology, Tianjin, 300308 China; 4 College of Biotechnology and Pharmaceutical Engineering, State Key Laboratory of Materials-Oriented Chemical Engineering, Jiangsu National Synergetic Innovation Center for Advanced Materials (SICAM), Nanjing Tech University, Nanjing, 211816 China

**Keywords:** Mannanase, Polyhydroxyalkanoates, Immobilization

## Abstract

Galactomannan oligosaccharides (GMOS), composed of 2–10 mannose units linked with β-1, 4 glycosidic bond as the main chain and galactose linked with α-1, 6 glycosidic bond as the side chain, are crucial for probiotic food synthesis due to their ability to promote the growth and activity of beneficial intestinal microbiota, enhance the host immune system, and improve nutrient digestion. GMOS is usually obtained by hydrolyzing plants such as locust bean gum and guar gum with mannanase. β-mannanase ManA from Alkaliphilic *Bacillus* sp. N16-5 can hydrolyze β-1, 4 glycosidic bond of galactomannan. In this study, an immobilization system was employed utilizing polyhydroxyalkanoate (PHA) biopolymers, which naturally have an affinity mainly mediated by hydrophobic interaction for PhaP protein. Fusion protein combining ManA with PhaP from *Aeromonas hydrophila*, was subsequently immobilized on PHA support to form a multi-enzyme complex, facilitating the hydrolysis of locust bean gum to generate GMOS. This immobilized enzyme enhances enzyme stability and reusability, can be reused up to 32 times while maintaining ~ 80% of its activity, offering substantial cost savings through in-situ enzyme and product separation. Additionally, the different PHA forms were developed to hydrolyze locust bean gum to produce GMOS, such as nano PHA particles, PHA electrospun materials, while these preliminary investigations show promise, further research is needed to optimize their performance and practical application.

**Graphical Abstract:**

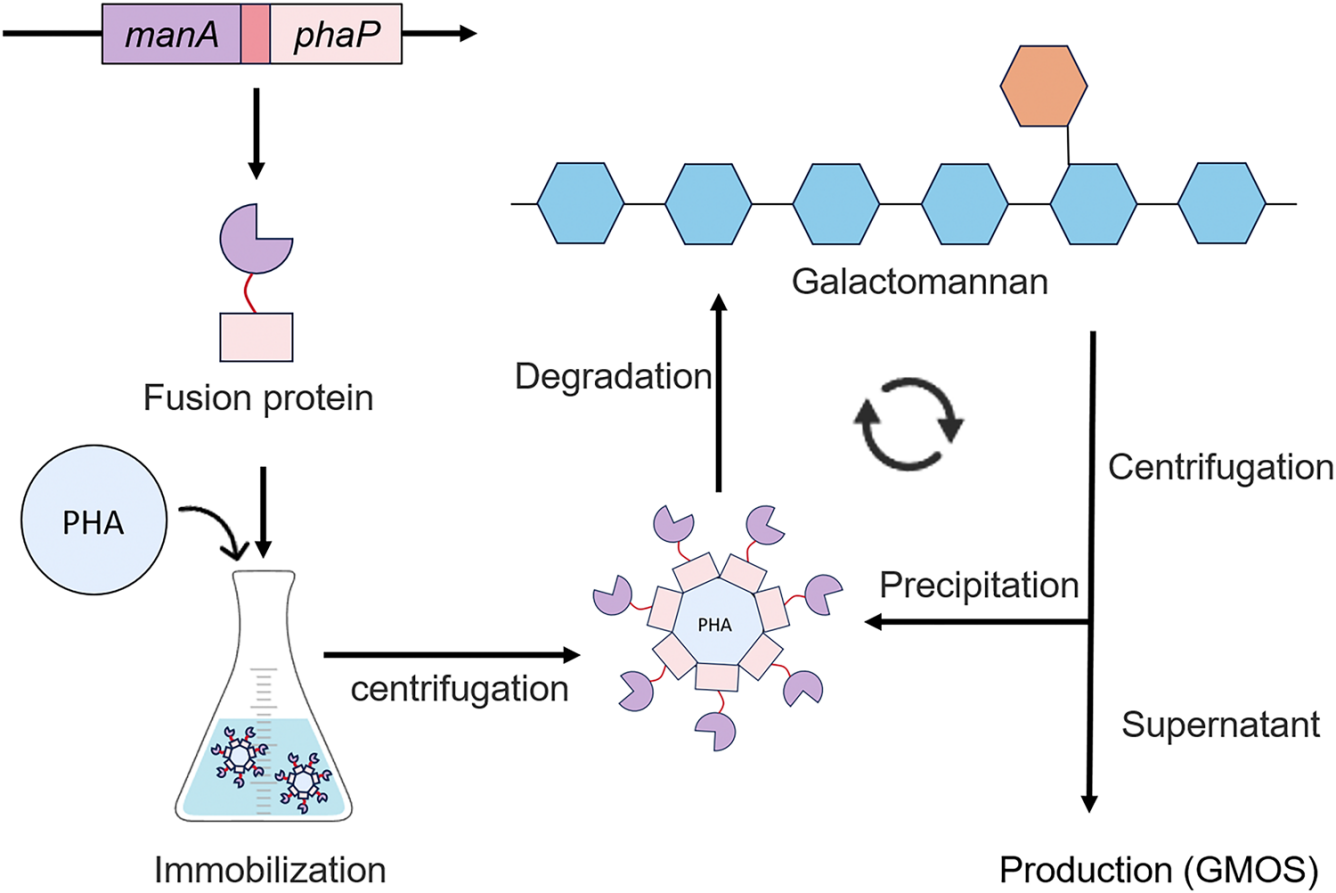

## Introduction

Mannanase (EC 3.2.1.78) is an important industrial enzyme that catalyzes reaction of mannan hydrolysis (Krylov et al. 2018) and recently more interesting reaction of transgalactosylation (Freiesleben et al. 2019), yielding a highly valuable group of prebiotic compounds named GMOS (Chacher et al. 2017; Miao et al. 2021). GMOS, composed of 2–10 galactose and mannose units linked by β-1,4-glycosidic bonds (Carević et al. 2018; Faustino et al. 2021; Nopvichai et al. 2019), are highly valued in probiotic food synthesis for promoting intestinal microbiota (Jana et al. 2020; Liu et al. 2019), enhancing the immune system (Guan and Li 2009; Li et al. 2023), and supporting overall animal growth (Forsatkar et al. 2018). In order to reduce the cost of preparing GMOS, immobilized enzymes that demonstrate good performance, require simple preparation, and are safe, inexpensive, and reusable must be developed urgently (Chen et al. 2023a; Nguyen et al. 2018).

Enzyme immobilization techniques involve covalent binding, encapsulation, entrapment, adsorption, etc. (Maghraby et al. 2023). Immobilization methods of β-mannanase are covalent method (Sadaqat et al. 2022; Anderson et al. 2022) and cross-linking method (Panwar et al. 2017; Behera et al. 2022). Traditional immobilization carriers for β-mannanase, such as chitosan beads (Sadaqat et al. 2022), calcium alginate (Chen et al. 2023b), ZnO (Dikbaş et al. 2023) and epoxy resin (Murillo-Franco et al. 2024), have been extensively explored. Despite their utility, these carriers often face limitations in cost, scalability, or reusability. An approach utilizing polyhydroxyalkanoates (PHA) and their associated binding protein, PhaP, offers a promising alternative. PHAs are biocompatible, bacterially produced polymers, serving as energy storage molecules in nutrient-scarce conditions (Mai et al. 2024). PhaP from *Aeromonas hydrophila *(Zhao et al. 2016), an amphiphilic protein, binds specifically to hydrophobic PHA surfaces via strong hydrophobic interactions (Lan et al. 2016; Wei et al. 2011). PhaP-mediated immobilization has been applied to enzyme immobilization. This hydrophobic interaction enables that enhance enzyme stability, simplify recovery, and improve reusability, thereby reducing industrial costs (Putri et al. 2023; Wang et al. 2023; Yushkova et al. 2019). For instance, The fusion of bacterial organophosphorus anhydride hydrolase with PhaP and its immobilization on PHA granules resulted in higher catalytic efficiency (Li et al. 2019). The fusion protein of PhaP and carbonyl reductase was immobilized on polyhydroxyalkanoate (PHA) magnetic microspheres, and the catalyst maintained 78.6% activity after 10 recovery cycles (Han et al. 2023a). Additionally, the specific binding affinity between PhaP and PHA granules has been exploited for targeted drug delivery applications. A tumor targeting system was developed by presenting an epidermal growth factor receptor (EGFR)-targeting peptide (ETP) on the surface of PHA NPs, via PhaP mediated adsorption (Fan et al. 2018). The ligand–PhaP–PHA specific drug delivery system was proven effective both in vitro and in vivo (Yao et al. 2008). PhaP-PHA immobilization strategy has not been applied to β-mannase yet. The extracellular endo-β-mannanase, ManA (GenBank accession no.AAT06599), encoded by the mannan utilization gene cluster was successfully characterized biochemically and structurally in the previous studies (Zhao et al. 2011, 2008; Ma et al. 2004). ManA degrades mannan into mannan oligosaccharides by cleaving β-1, 4 glycosidic bonds (Song et al. 2018). The objective of this study is to realize the recycling of immobilized enzyme to hydrolyze locust bean gum to generate GMOS.

In this study, ManA was fused with PhaP and immobilized on PHA support. The enzymatic activity retention of the immobilized ManA-PhaP@PHA system was then investigated over multiple reuse cycles. Fluorescence microscope was used to characterize the adsorption effect of PHA particles on EGFP-PhaP. However, a key challenge with PHA-based immobilization is the large size of PHA particles, which typically exhibit low specific surface areas and limited enzyme adsorption capacity. PHA particles smaller than 1 µm (nanoparticles) or within the 200 nm to 100 µm range (micro/nanoparticles) offer greater surface area for enzyme immobilization (Han et al. 2023b; Jang et al. 2024). To address this, nano-scale PHA particles and electrospun PHA materials were explored to hydrolyze locust bean gum to produce GMOS.

## Results and discussion

### Cloning and expression of fusion protein

**Fig. 1:**
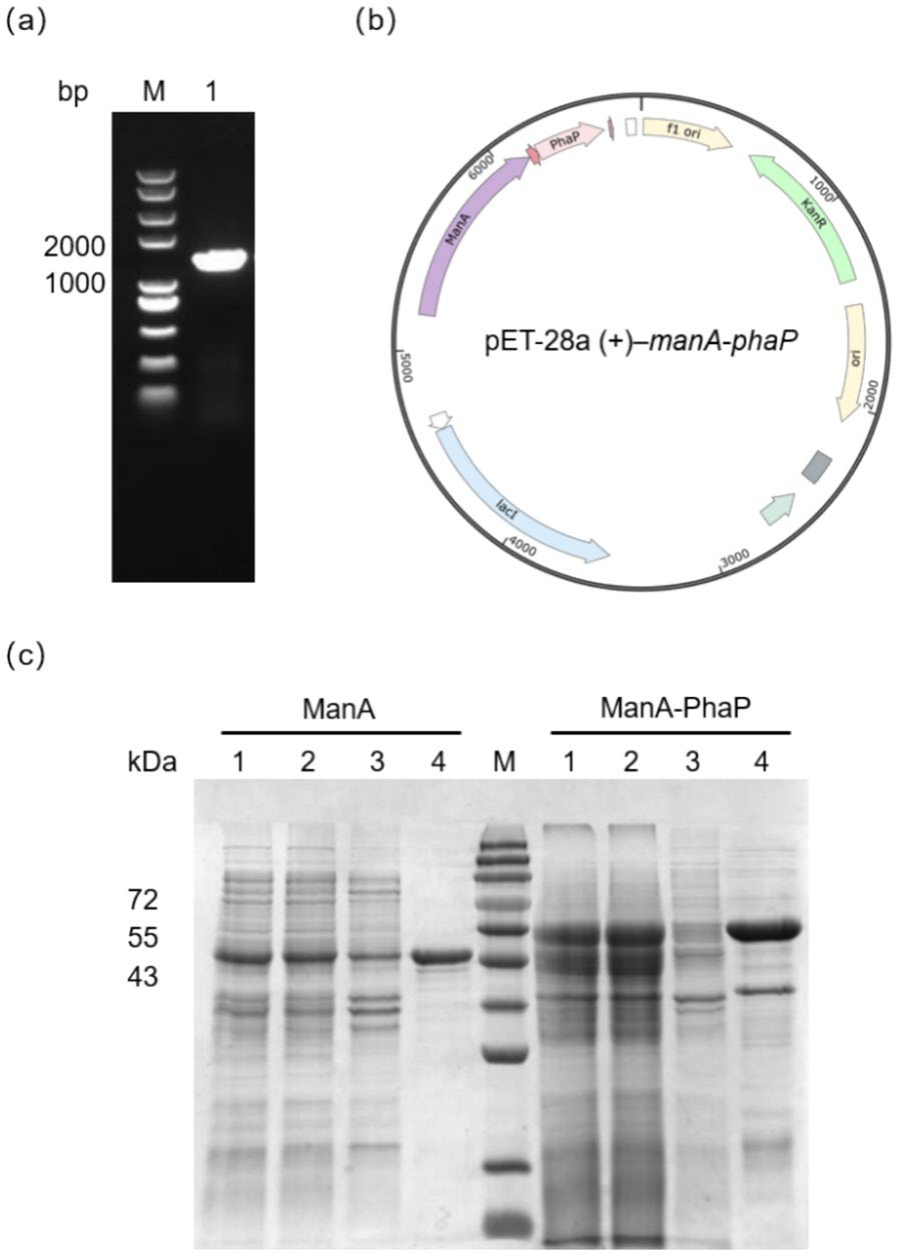
Gene cloning and expression of fusion protein. **a** PCR products *manA-phaP* generated by the overlap extension PCR. Lane M: DNA marker; **b** Map of the vector pET28a (+) –*manA*-*phaP*; **c** SDS-PAGE analysis of the expression and purification of PhaP-fusion mannanase. Lane M: protein marker. Lane 1: total protein extract; Lane 2: soluble protein extract; Lane 3: insoluble protein extract; Lane 4: NTA-purified protein

A 969 bp *manA* gene and a 348 bp *phaP* fragment have been successfully obtained and the *manA-phaP* fragment was obtained by overlapping extension (Fig. 1a). Insert the fragment was inserted into the pET-28a (+) vector by overlap extension, yielding a recombinant protein expression vector, pET-28a (+)–*manA*-*phaP* (Fig. 1b). Under optimal conditions, the target recombinant protein was highly soluble. No obvious protein bands were observed in the pellets of the *E. coli* cell lysate, suggesting that no ManA-PhaP inclusion bodies were formed. The protein was purified based on its His-tag, as shown in Fig. 1c, resulting in the purified fusion protein ManA-PhaP. It was deduced from SDS-PAGE that ManA was a protein with a molecular weight of 44 kDa, and ManA-PhaP was a protein with a molecular weight of 54.5 kDa.

### PHA supports materials

**Fig. 2:**
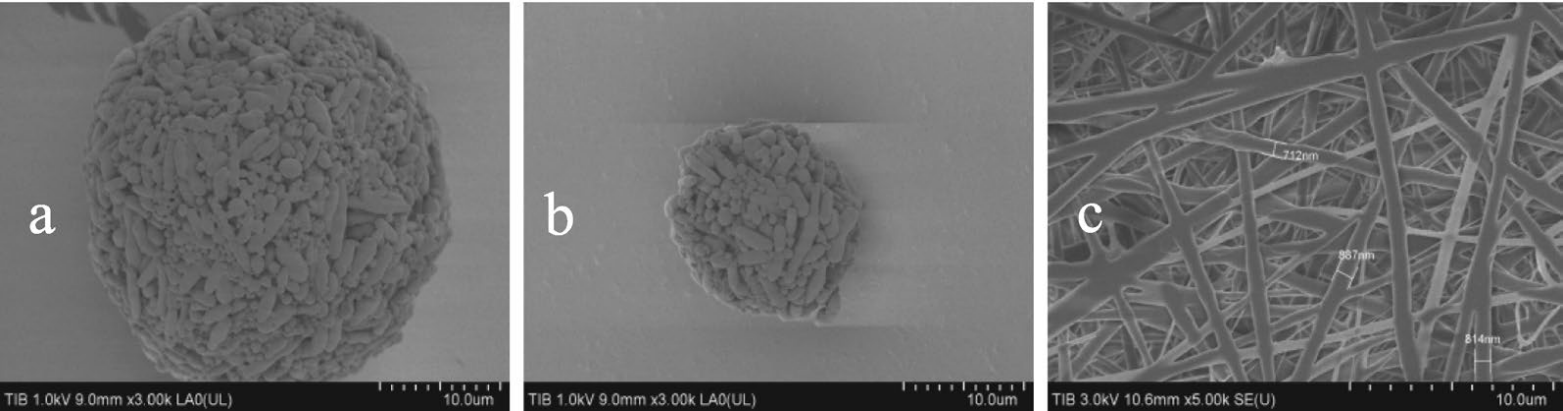
PHA supports materials. **a** PHA granules under SEM; **b** PHA nanoparticles under SEM; **c** Nanofibers under SEM

To enhance the surface area-to-volume ratio, PHA nanoparticles and PHA nanofibers were prepared, and examined the details using microscopy and scanning electron microscopy (SU8010, Hitachi, Japan). As shown in the following figures, both nanoparticles and nanofibers were successfully fabricated. The nanoparticles (Fig. 2b) were smaller than PHA granules (Fig. 2a). The diameter of the nanofibers was smaller than 1 µm (Fig. 2c). This demonstrates that a new type of PHA material has been successfully created.

### Immobilized fusion protein

**Fig. 3:**
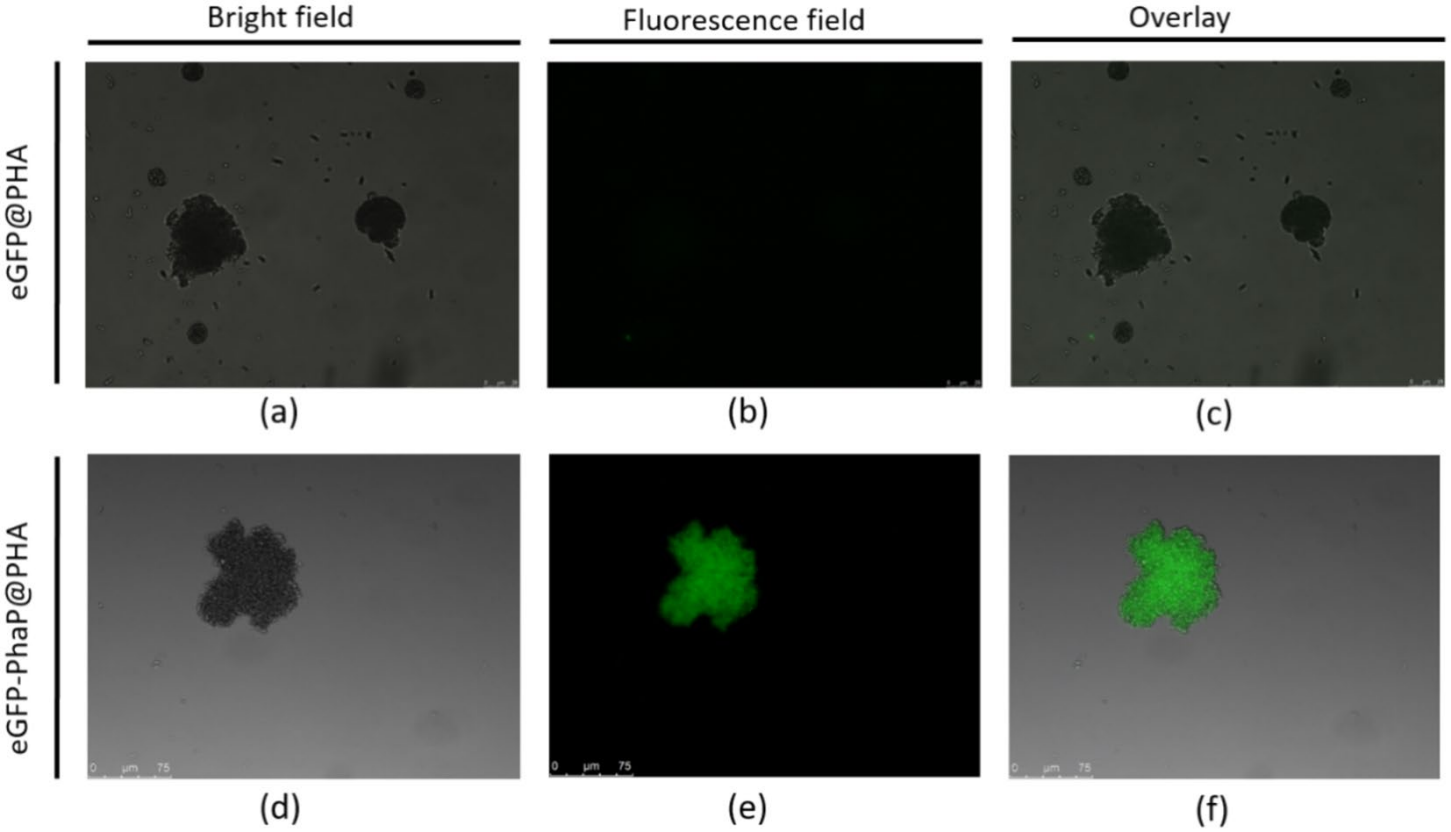
Immobilization of eGFP-PhaP with PHA supports

The green fluorescent protein (eGFP) was genetically fused with the binding protein PhaP and expressed as a fusion protein. After mixing the eGFP-PhaP fusion protein with PHA supports for an adequate time, extensive washing with a buffer solution was performed to eliminate non-specifically bound proteins. Examination under a fluorescence microscope revealed that the eGFP protein without the PhaP binding protein did not exhibit fluorescence, whereas eGFP-PhaP displayed a distinct fluorescence signal within the immobilized area (Fig. 3). This indicates effective and specific adsorption of the fusion protein onto the PHA supports.

**Fig. 4:**
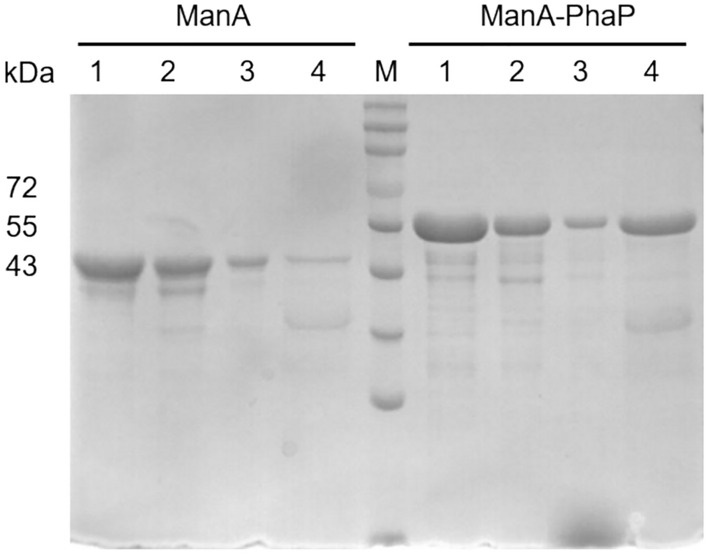
SDS-PAGE analysis of immobilized enzyme wash. Lane M: protein marker. Lane 1: purified protein; Lane 2: non-binding protein in reaction supernatant; Lane 3: buffer wash of immobilized protein; Lane 4: 10% SDS solution wash of immobilized protein

SDS-PAGE analysis of the immobilized enzyme wash demonstrates that while the buffer solution cannot remove the enzyme from PHA supports, a 10% SDS solution can effectively elute the specifically bound enzyme from the PHA supports. This finding illustrates that the immobilized enzyme exhibits specific adsorption to PHA materials (Fig. 4).

### Properties of immobilization enzyme

**Fig. 5:**
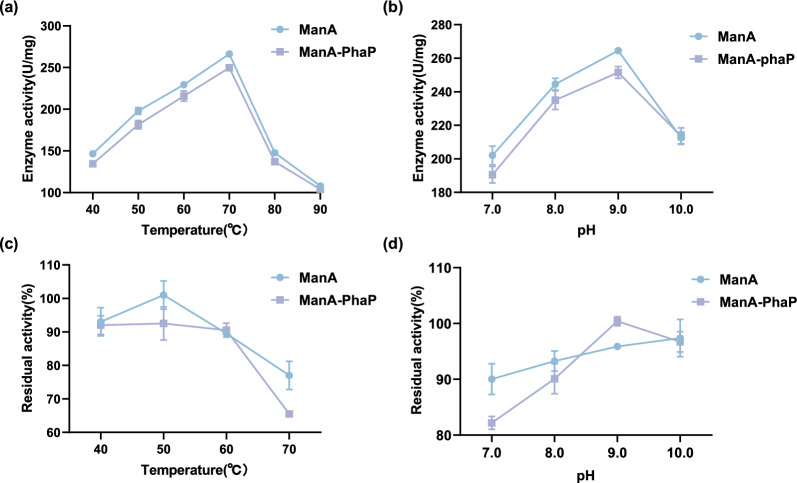
Enzymatic properties of ManA and ManA-PhaP. **a** Effect of temperature on enzyme activity; **b** Effect of pH on enzyme activity; **c** Residual enzyme activity at different temperatures; **d** Residual enzyme activity at different pH

A comparative analysis of the immobilized and free enzymes was conducted using the DNS assay. The optimal reaction pH of ManA-PhaP for the hydrolysis of LBG was 9.0. The optimal reaction temperature was 70  C, which is the same as that for free ManA. This indicates that the presence of PhaP in the fusion protein does not interfere with biochemical characteristics. Additionally, ManA-PhaP can maintain 100% relative activity at pH 9.0 for 150 min and 90% relative activity at 50–60 °C for 150 min (Fig. 5), demonstrating significant potential for applications in enzyme immobilization compared to the free enzyme. Furthermore, the immobilized enzyme exhibited slightly lower activity than the free enzyme under optimal conditions (Fig. 5a, b). The enzyme activity is sometimes slightly lost after immobilization. An enzyme cocktail with β-mannanase as the main activity was immobilized on epoxy resin foams filled with fibers from annatto capsules. The immobilized enzyme exhibited an activity retention of 79.61% compared to the free enzyme (Murillo-Franco et al. 2024). This will guide the optimization of future immobilization strategies to enhance the enzyme’s activity and stability.

### Reuse of immobilization enzyme

**Fig. 6:**
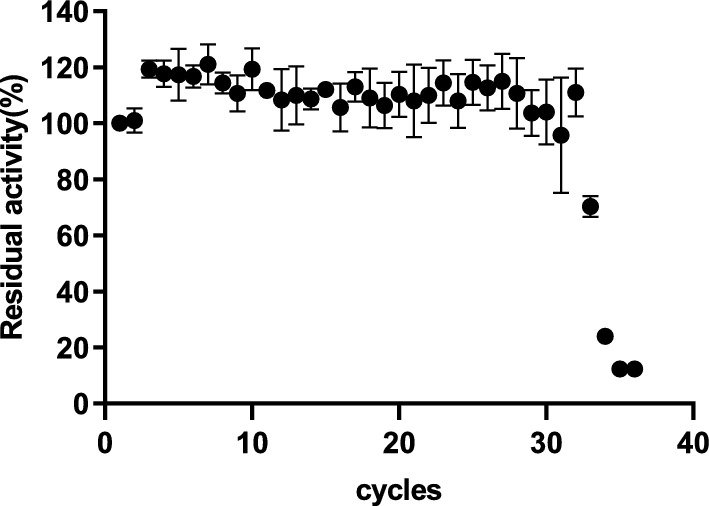
Reusability of immobilized enzyme

To evaluate the effects of enzyme immobilization, the enzyme activity in the hydrolysis of locust bean gum (LBG) was analyzed. All analyses were carried out in triplicate. The enzyme activity was measured using the DNS standard assay procedure. Immobilized enzymes were reused in hydrolysis reactions over 48 h, and the supernatant, which was centrifuged from the reaction, could contain 80% residual enzymatic activity after recycling 32 reactions (Fig. 6). This high residual activity underscores the efficiency of the separation and recycling processes, highlighting the robustness and reusability of the immobilized system. Comparative studies further contextualize these findings. For instance, β-mannanase immobilized on sodium alginate grafted with β-cyclodextrin retained 70% of its activity after 15 reuse cycles. (Dhiman et al. 2020). β-Mannanase was immobilized on calcium alginate beads, which retained 70.34% of its activity after 8 reuse times (Chen et al. 2023b). *Endo*-β-1, 4-mannanase (ManB-1601) was first immobilized using cross linked enzyme aggregates and later for further improvement in properties along with facile and energy efficient separation was grafted on to chitosan magnetic nanocomposites. This system sustained hydrolysis of locust bean gum for 12 cycles (Panwar et al. 2017).

### Preparation oligosaccharides from LBG by immobilized enzyme

**Fig. 7:**
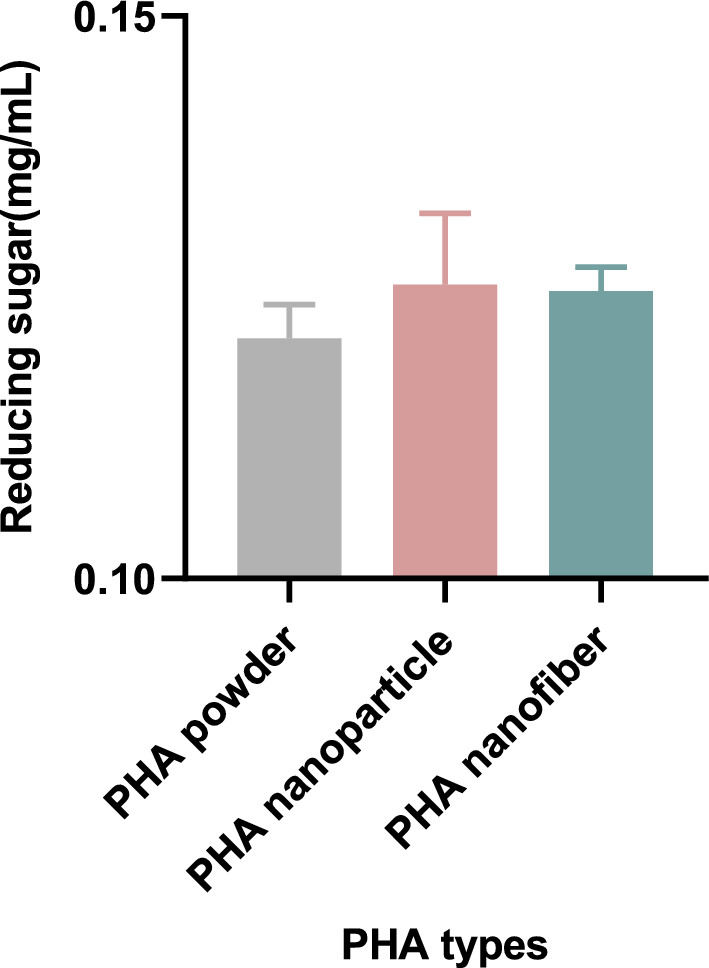
Reducing sugar released by different types of PHA

The immobilization of enzymes using different types of PHA materials and the determination of oligosaccharides in the reaction are shown in Fig. 7. The results indicate that the reducing capacity of PHA nanoparticles is slightly stronger than that of the PHA supports (PHA powder). Nanoparticle adhesion benefits from a large surface area, significantly increasing contact with substrates as particle sizes decrease (Jang et al. 2024; Kim et al. 2017). In contrast, the reducing capacity of PHA nanofibers is weaker, which may be due to the stronger hydrophobicity of the nanofibers, making them less conducive to immobilization with enzyme solutions and resulting in lower immobilization efficiency.

## Conclusion

The mannanase fused with the PhaP binding-protein module can specifically adsorb onto PHA supports. The immobilized enzyme can maintain its enzymatic properties and retain 80% relative activity after being reused for 32 cycles, which is significant for the commercial industry. Additionally, the combined use of PHA materials with immobilized enzymes allows for simple separation of enzymes and products through a one-step centrifugation process. Furthermore, it can be combined with an ultrafiltration system to obtain polysaccharide products of different molecular weights, which holds great potential for application. This study confirmed the potential of PHA as an immobilized material, but its long-term stability, large-scale production and biocompatibility need to be further verified. In the future, the application value of PHA in immobilization technology can be further enhanced by functional modification and optimization of sustainable production process.

## Materials and methods

### Cloning and expression of fusion protein

The target gene was obtained from our previous research (Zhao et al. 2008), which is cloned from alkaliphilic *Bacillus* sp. N16-5. The PhaP sequence was synthesized by Genewiz Corporation (Soochow, China). The target fragments were amplified via overlap and then cloned into the pET-28a (+) vector. The verified correct vector, pET-28a (+)-*manA*-*phaP*, was transformed into *E. coli* BL21(DE3). The strain was precultured in 100 mL of LB medium containing 50 μg/mL kanamycin in a 500 mL shake flask and shaken at 220 rpm at 37 ℃ until the OD_600_ reached 0.6 ~ 0.8. Isopropyl β-D-thiogalactoside was then added to a final concentration of 0.1 mM. The cells were cultured at 25 ℃ for an additional 16 h. Cells were harvested by centrifugation at 4 ℃ and 8000 rpm for 20 min. The harvested cells were then ultrasonicated on ice. The recombinant proteins were purified by Ni–NTA resin. The homogeneity of the proteins was checked using 12% SDS-PAGE. Protein concentrations were determined by the BCA Protein Assay Kit (Solarbio, China) with bovine serum albumin as the standard.

### Preparation of PHA nanoparticle

Dissolve PHA powder in dichloromethane (10%) until a uniform liquid is formed. Gradually add this solution drop by drop into 100 mL of 1% polyvinyl alcohol (PVA) and stir at high speed overnight. Evaporate the dichloromethane at 45 °C in a fume cupboard, centrifuge at 8000 rpm for 30 min, and discard the supernatant. Wash the precipitate extensively with water to remove residual PVA, then freeze-dry to obtain PHA nanoparticles.

### Preparation of PHA nanofibers

Dissolve PHA in trifluoroethanol (20%) until a uniform liquid is formed, resulting in a PHA spinning solution. Transfer the PHA spinning solution to a 10 mL syringe and secure it onto the syringe pump of the spinning machine (YFSP-B01, Yunfan Technology, China), which should be equipped with a #30 stainless steel needle and a compatible airflow spinning nozzle. Connect the needle to the positive terminal of a high-voltage power supply and the receiving roller to the negative terminal. Set the syringe pump to a specification of 10 mL, with a supply rate of 0.008 mm/s, a nozzle gas pressure ranging from 0.01 to 0.02 MPa, a roller speed of 3000 rpm, and a spinning voltage of 20 kV.

### SEM characterization

SEM (SU8010, Hitachi, Japan) were used to observe the morphology and size of PHA materials. The samples were mounted onto the SEM stage. Subsequently, gold sputtering was performed using an ion sputter (E-1045, Hitachi, Japan) at a current of 15 mA for 120 s. The sputtered samples were then introduced into the SEM for observation. The morphologies of PHA powders and PHA nanoparticles were examined at 3000 × , and their diameters were measured. Meanwhile, the PHA fibers were observed at 5000 × , and the fiber diameters were determined.

### Preparation of immobilized enzyme

Mix the purified fusion protein as presented in the Cloning and Expression of Fusion Protein Sect. (5 mg) with the PHA material (500 mg) thoroughly. Incubate the mixture at 37 °C for 1 h or overnight at 4 °C. Then, centrifuge to obtain the immobilized mannanase on the PHA material. Wash the immobilized mannanase with excess buffer several times to remove any non-specifically bound proteins. Subsequently, wash the immobilized mannanase with a 10% SDS solution to verify specifically bound enzymes. The amount of enzyme immobilized on PHA was obtained by calculating the difference between the total protein amount and the free protein amount in the immobilized system.

### Enzyme properties of immobilized enzyme

The optimal pH for enzyme activity was studied using the following buffer system (100 mM): K₂HPO₄-KH₂PO₄ (pH 7.0), Tris–HCl (pH 8.0–9.0), and Gly-NaOH (pH 10.0). To determine pH stability, the enzyme was pre-incubated in buffers of different conditions for 24 h at 4 °C. Residual activity was measured using the DNS standard assay procedure. The optimum temperature for the enzyme was determined by measuring enzyme activity within the range of 40–90 °C. For thermostability, the enzyme was incubated at different temperatures (40–70 °C) for 150 min, followed by measuring residual activity using the DNS standard assay procedure.

One unit of β-endomannanase was defined as the amount of enzyme required to release 1 µmol of reducing sugar equivalent to mannose in 1 min at 50 °C. The free enzyme reaction activity is defined as 100%, under the optimal condition of 50 °C, pH 9.0, and enzyme activities under other conditions are measured and calculated relative to this standard. All experiments were conducted in triplicate.

### Hydrolysis to oligosaccharides from LBG by immobilized enzyme

In a 3 mL reaction system, mix 1.5 mL of 5% (w/v) LBG with 1.5 mL of the immobilized enzyme obtained previously. Initiate the reaction in a water bath at 40 °C for 8 h. Afterward, centrifuge the mixture at 12,000 rpm. The amount of oligosaccharide was calculated using the DNS reducing sugar determination method, with mannose as the standard equivalent to reducing sugar.

## Data Availability

All data generated or analysed during this study are included in this published article.

## Abbreviations

PHA: Polyhydroxyalkanoate
LBG: Locust bean gum
GMOS: Galactomannan oligosaccharides
SDS-PAGE: Sodium dodecyl sulfate–polyacrylamide gel electrophoresis
SEM: Scanning electron microscopy
DNS: 3, 5-Dinitrosalicylic acid
